# Multiple machine-learning tools identifying prognostic biomarkers for acute Myeloid Leukemia

**DOI:** 10.1186/s12911-023-02408-9

**Published:** 2024-01-02

**Authors:** Yujing Cheng, Xin Yang, Ying Wang, Qi Li, Wanlu Chen, Run Dai, Chan Zhang

**Affiliations:** Department of blood transfusion, The First People’s Hospital of Yunnan Province. The Affiliated Hospital of Kunming University of Science and Technology, No.157 Jinbi Road, 650034 Kunming, Yunnan, China

**Keywords:** Acute Myeloid Leukemia, Machine learning methods, LASSO, RF, SVM-RFE, eXtreme gradient boosting, Pan-cancer

## Abstract

**Background:**

Acute Myeloid Leukemia (AML) generally has a relatively low survival rate after treatment. There is an urgent need to find new biomarkers that may improve the survival prognosis of patients. Machine-learning tools are more and more widely used in the screening of biomarkers.

**Methods:**

Least Absolute Shrinkage and Selection Operator (LASSO), Support Vector Machine-Recursive Feature Elimination (SVM-RFE), Random Forest (RF), eXtreme Gradient Boosting (XGBoost), lrFuncs, IdaProfile, caretFuncs, and nbFuncs models were used to screen key genes closely associated with AML. Then, based on the Cancer Genome Atlas (TCGA), pan-cancer analysis was performed to determine the correlation between important genes and AML or other cancers. Finally, the diagnostic value of important genes for AML was verified in different data sets.

**Results:**

The survival analysis results of the training set showed 26 genes with survival differences. After the intersection of the results of each machine learning method, DNM1, MEIS1, and SUSD3 were selected as key genes for subsequent analysis. The results of the pan-cancer analysis showed that MEIS1 and DNM1 were significantly highly expressed in AML; MEIS1 and SUSD3 are potential risk factors for the prognosis of AML, and DNM1 is a potential protective factor. Three key genes were significantly associated with AML immune subtypes and multiple immune checkpoints in AML. The results of the verification analysis show that DNM1, MEIS1, and SUSD3 have potential diagnostic value for AML.

**Conclusion:**

Multiple machine learning methods identified DNM1, MEIS1, and SUSD3 can be regarded as prognostic biomarkers for AML.

**Supplementary Information:**

The online version contains supplementary material available at 10.1186/s12911-023-02408-9.

## Backgrounds

Acute myeloid leukemia (AML) is a malignant bone marrow disease characterized by clonal expansion and differentiation arrest of bone marrow progenitor cells. Most AML cases still have no clear etiology [1]. AML is the most common acute leukemia in adults, and its survival time is short [2]. In recent years, with the rapid development of molecular targeted therapy and combined therapy, and the widespread application of these two therapies in clinical practice, the survival and prognosis of AML patients have been relatively prolonged and improved [3]. Intensive chemotherapy and gene stem cell transplantation are usually applied to a small number of young patients, and for most patients, the prognosis and survival rate are poor [4]. Although the treatment strategies for AML have been continuously adjusted and improved over the past few decades, the effect of these treatment strategies on the survival and prognosis of patients is still minimal [5]. Therefore, the identification of new and effective prognostic biomarkers is crucial for accurately predicting the prognosis of AML patients and for a deeper and more comprehensive understanding of the pathogenesis of AML.

With the advancement of gene sequencing technology, a series of gene databases have emerged, such as the Cancer Genome Atlas (TCGA) and the Gene Expression Omnibus (GEO). In addition, machine learning algorithms, as one of the main tools of data mining, are now widely used in the medical field. The algorithm establishes a risk model by learning the existing data of patients, which is used to predict the disease, diagnose the severity of the disease, and evaluate the prognosis of the disease [6, 7]. Its main types include the least absolute shrinkage and selection operator (LASSO), random forest graph (RF), support vector machine (SVM), decision tree, and other common algorithms. LASSO is the only property of the absolute value of the penalized regression coefficient [8]. The greater the penalty, the greater the shrinkage of the coefficient, and then remove the unimportant covariates [9, 10]. Support vector machine recursive feature elimination (SVM-RFE) is a supervised machine learning technique widely used in classification and regression. Its purpose is to classify data points by maximizing the margin between classes in high-dimensional space. The features are classified according to the accuracy value, and several features with higher accuracy are selected [11]. The RF algorithm is a method of training and predicting samples by constructing a decision tree. The features with high importance scores are obtained by calculating and sorting the importance scores of features [12]. These machine algorithms can learn and train from data to achieve accurate predictions of future events [13]. These algorithms are gradually being used in the prognosis of lung cancer, breast cancer, liver cancer, gastrointestinal cancer, and other malignant tumors, which has become a hot spot in clinical research [14–17].

Machine learning algorithms contain a variety of types, and each model has its scope of application. Different types, different volumes, and different characteristics of data have different prediction performance. Therefore, this study aims to screen genes related to AML prognosis based on multiple types of machine learning, and then take the intersection gene of each machine learning result as the key gene for subsequent research. Finally, pan-cancer analysis was performed on key genes to further clarify the correlation between key genes and the occurrence and development of AML or other cancers and then to clarify the diagnostic value of key genes for AML. This study will provide new ideas for the prognosis evaluation of AML patients, and then promote the efficient and accurate individualized treatment of AML.

## Methods

### Acquisition of data sets

Data on acute myeloid leukemia were obtained from the Gene Expression Omnibus (GEO) database (https://www.ncbi.nlm.nih.gov/geo/query/acc.cgi). Due to the excessive amount of search data, we set some conditions to select candidate data sets. The specific screening criteria for the dataset used in this study were acute leukemia, United States, Homo sapiens, adults, and with a total sample size greater than 100 people. Finally, three data sets were selected for subsequent research (GSE63270, GSE15061, and GSE48558). The basic information of each data set is shown in Table 1. The gene set of GSE15061 was obtained for expression difference analysis (setting the threshold: |logFC| > 1.5, *P* < 0.05). The differential volcano map (http://sangerbox.com/tool.html) was drawn using the SangerBox website.

**Table 1:** Basic information for all data sets used in this study

Data sets ID	Platforms	Samples		Application
		Control	AML cases	
GSE15061	GPL570	138	404	Test set
GSE63270	GPL17810	42	62	Verification set
GSE48558	GPL6244	49	39	Verification set

AML: acute myeloid leukemia

### Screening the key genes related to the prognosis of AML based on multiple machine-learning methods

Kaplan-Meier (KM) survival analysis of differentially expressed genes in the training set was performed using the R-4.1.1 software package (“survival” and “survminer”).

Subsequently, R-4.1.1 different software packages were used to perform machine learning such as LASSO (R package: “glmnet”, “survival”, “survminer”), RF (R package: “randomForest”, “caret”, “varSelRF”), SVM-RFE (R package: “svm”, “caret”, and “randomForest”), and XGBoost (R package: “xgboost”, and “caret”) on genes related to AML prognosis in the training set. In this study, we using the different functions in caret package of the R software (lrFuncs, IdaProfile, caretFuncs, and nbFuncs) to screened the optimal genes. The specific operation process and screening criteria were carried out according to the official manual of R software (http://topepo.github.io/caret/recursive-feature-elimination.html#recursive-feature-elimination-via-caret). The parameters of the machine learning algorithms used in this study were set according to previous studies [18–20].

Finally, the key genes were obtained after the intersection of the genes screened by the above machine-learning methods for subsequent analysis.

### Pan-cancer analysis of key genes

Using the UCSC XENA database (http://xenabroswer.net/hub), which integrates public data from multiple databases, downloaded data sets that have been uniformly standardized (including the Cancer Genome Atlas (TCGA), Genotype Tissue Expression (GTEx). We extracted the expression of key genes in 33 cancer types, immune subtypes, clinical information, and other data from the downloaded data set. Then SangerBox (http://sangerbox.com/tool.html) was used to analyze the expression of key genes in cancer, survival analysis, immune analysis, and so on.

The correlation matrix heat map was drawn by SangerBox to explore the expression differences at immune checkpoints. The box plot was drawn by the R package (“ggplot2”, “ggsignif”, “ggpubr”, and “RColorBrewer”) to analyze the correlation between key genes and different subtypes of immune cells.

### Gene set enrichment analysis (GSEA) of key genes

To identify pathways associated with key genes, we performed GSEA enrichment analyses. GSEA does not require genetic screening, thereby preserving genes that are not significantly different in expression but are functionally important [21]. GSEA analysis was performed by Sangerbox online bioinformatics tool (http://sangerbox.com/tool.html) based on AML mRNA data in the training set (GSE15061), which will identify the signaling pathways that potentially be related to the key genes screened by the machine learning algorithm.

### Validation of diagnostic value of key genes

To verify the diagnostic value of key genes for acute myeloid leukemia, GSE63270 and GSE48558 data sets were used for verification. The “barplot” software package in R was used to verify the differential expression of key genes in AML patients, and the ROC (receiver operating characteristic curve) curve was drawn to evaluate the diagnostic value of key genes.

## Results

### Survival analysis of differentially expressed genes

**Fig. 1:**
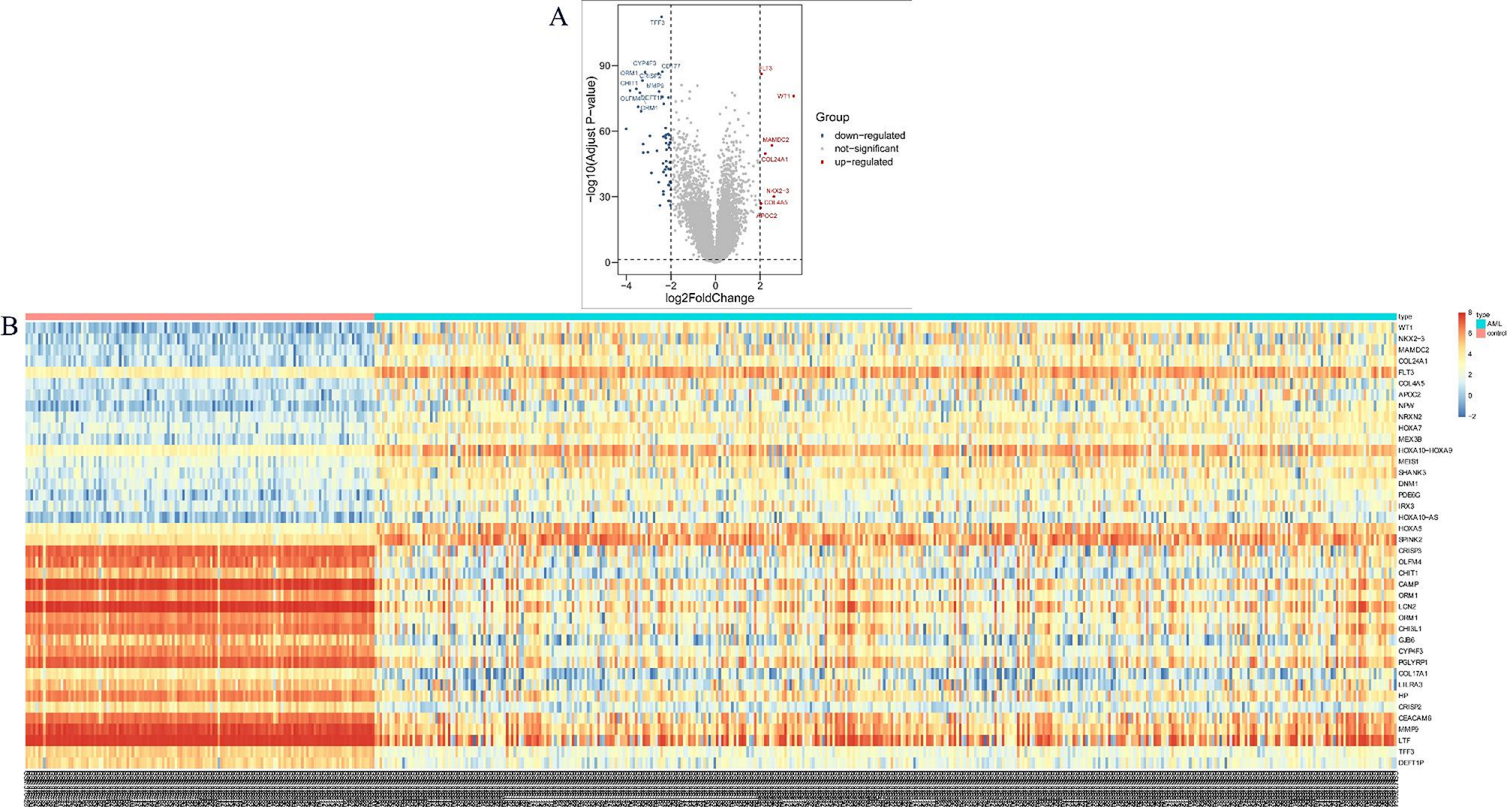
Differentially expressed genes in training set GSE15061. (**A**) Volcano maps showed the expression patterns of differentially expressed genes in the training set; (**B**) Heat maps of differentially expressed genes in the training set

The difference expression analysis of the training set GSE15061 was performed, and the threshold was set: |logFC| > 1.5, *P* < 0.05. The results showed that (Fig. 1) there were 171 differentially expressed genes, including 151 down-regulated genes and 20 up-regulated genes. Subsequent survival analysis of differentially expressed genes showed that a total of 26 differentially expressed genes had prognostic differences in the training set (Supplemental Fig. 1).

### Screening key genes related to AML prognosis based on multiple machine learning

**Fig. 2:**
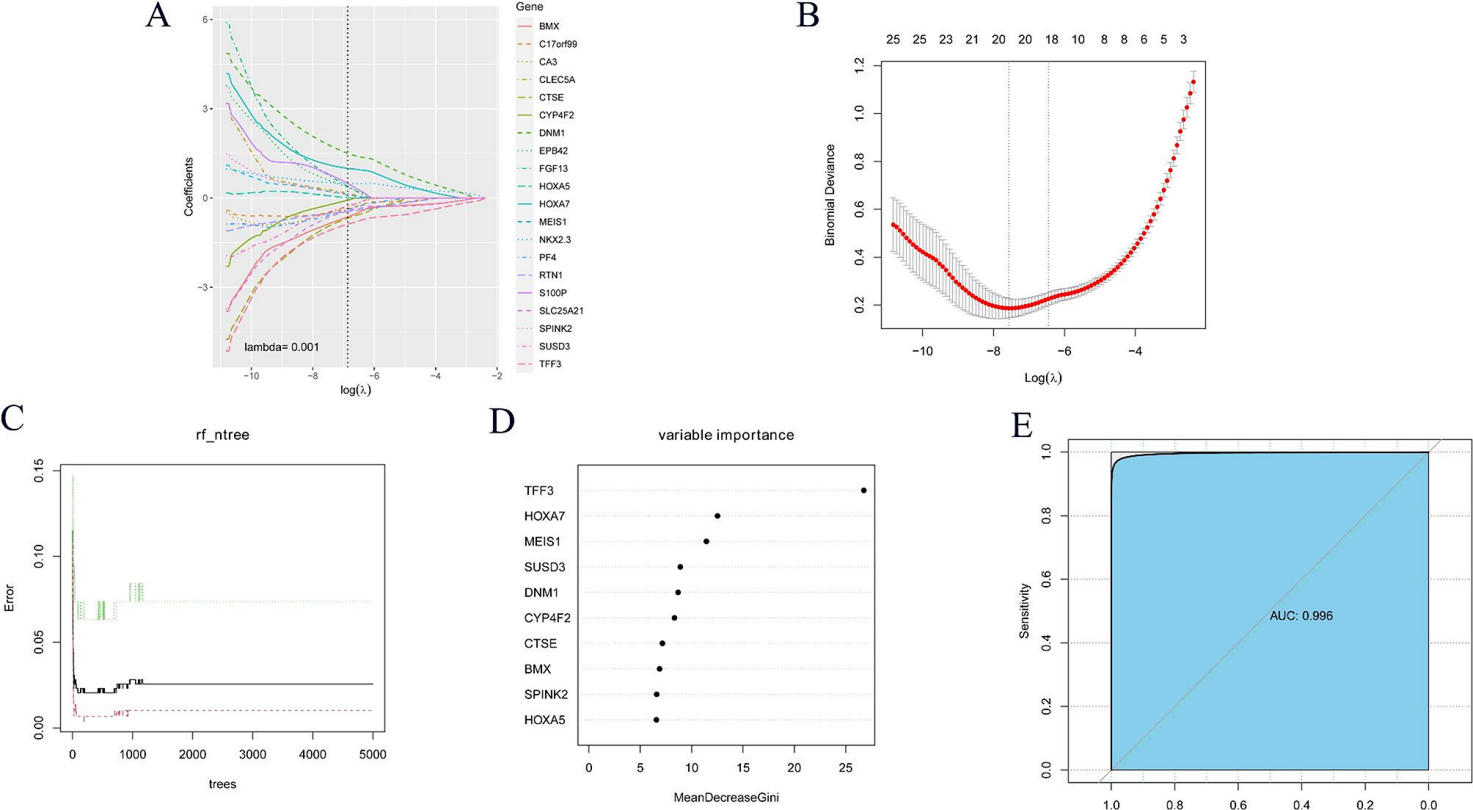
Screening important genes related to the prognosis of AML patients based on LASSO and RF models. (**A**) and (**B**) indicate that LASSO (least absolute shrinkage and selection operator) screened 20 important genes associated with AML prognosis. The method uses an L1 penalty to shrink some regression coefficients to exactly zero. (**A**) Ten time cross-validation for tuning parameter selection in the LASSO model; The binomial deviance curve was plotted versus log (λ), where λ is the tuning parameter. (**B**) LASSO coefficient profiles; LASSO coefficient profiles of clinic pathologic variables. (**C**), (**D**) and (**E**) indicate that the RF (random forest) algorithm screened the top five genes ranked by importance, which were related to AML prognosis. (**C**) The effect of the number of decision trees on the error rate (when the number of decision trees is about 2000, the error rate is relatively stable); The x-axis represents the number of decision trees and the y-axis represents the error rate. (**D**) Gini coefficient method in random forest classifier. x-axis: the genetic variable; y-axis: the importance index. (**E**) The ROC curve of the RF model, The AUC (area under the ROC curve) value is 0.977, which indicates that the predictive performance of the RF model is good

**Fig. 3:**
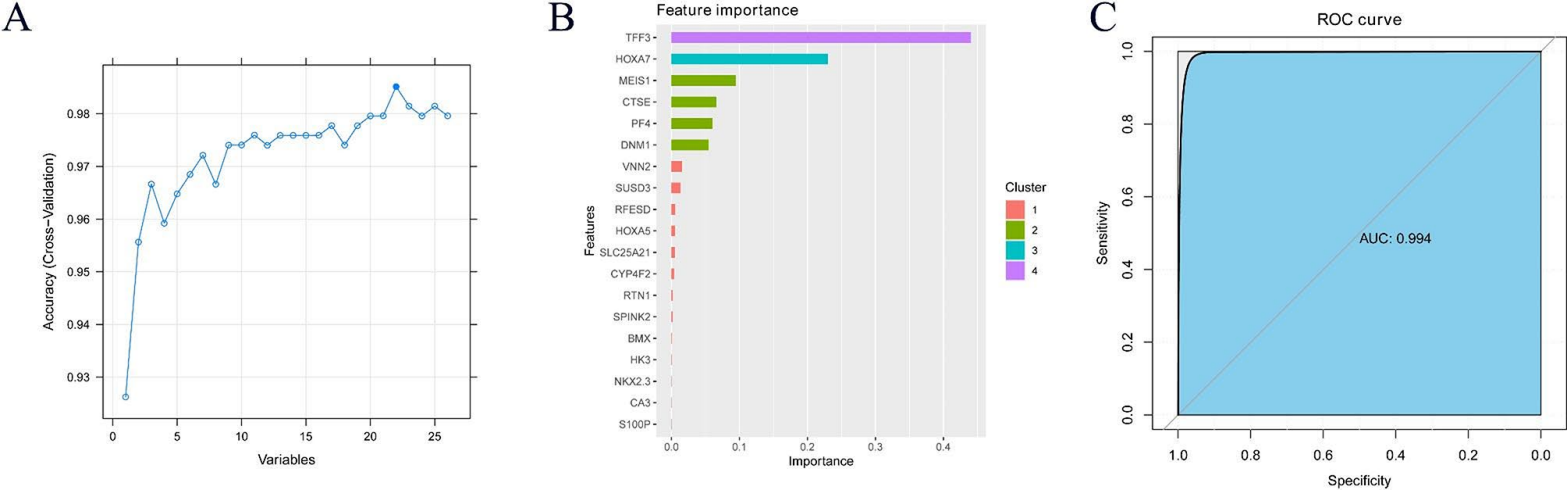
Screening important genes associated with the prognosis of AML patients based on SVM-RFE and XGBoost models. (**A**) indicates that the SVM-RFE algorithm identified 22 important genes. The SVM-RFE algorithm filtered 26 genes with prognostic value to determine the best combination of feature genes. Finally, 22 genes (maximum accuracy = 0.9797) were identified as the optimal feature genes. (**B**) and (**C**) indicate that the XGBoost algorithm identified 19 important genes. (**B**) Importance scores of the top 19 important genes and corresponding variables screened by XGBoost. X-axis indicates the importance score which is the relative number of a variable that is used to distribute the data, Y-axis indicates the top 19 weighted variables (**C**) The ROC curve of the XGBoost model, The AUC (area under the ROC curve) value is 0.964, which indicates that the predictive performance of the XGBoost model is good

**Fig. 4:**
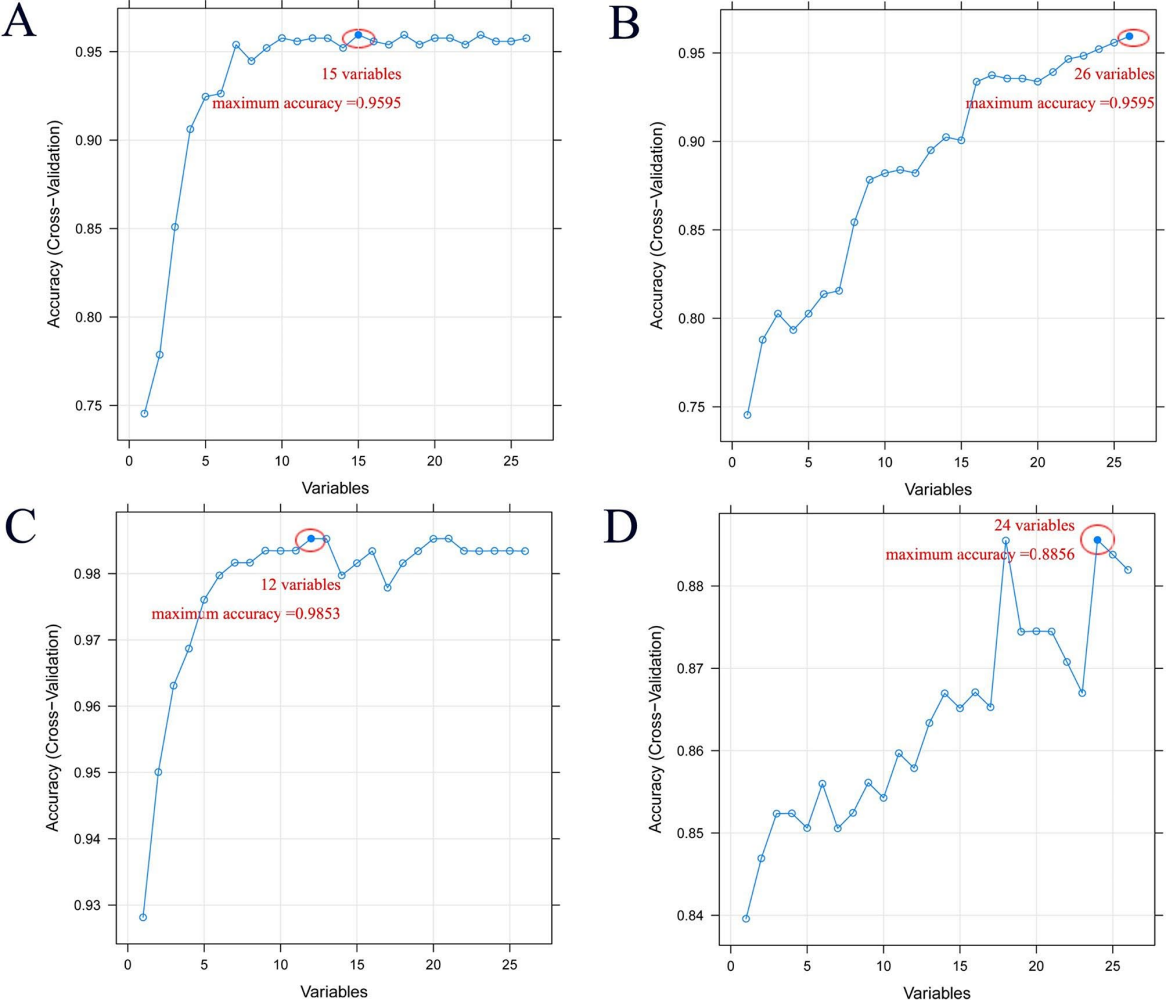
Screening important genes related to the prognosis of AML patients based on recursive feature elimination (RFE) algorithm. Feature selection is performed using multiple functions in the R package caret (lrFuncs, IdaProfile, caretFuncs, and nbFuncs). (**A**) lrFuncs model identified 15 genes as the optimal characteristic genes (maximum accuracy = 0.9595). (**B**) 26 genes were identified as the optimal feature genes by the IdaProfile model (maximum accuracy = 0.9595); (**C**) caretFuncs model identified 12 genes as the optimal characteristic genes (maximum accuracy = 0.9853); (**D**) nbFuncs model identified 24 genes as the optimal feature genes (maximum accuracy = 0.8856)

Firstly, LASSO regression analysis was used to screen 26 genes with prognostic value, and 10-fold cross-validation was performed. According to LASSO regression machine learning (Fig. 2A–B), a total of 20 genes were screened. Then, we use the RF algorithm (parameter settings: ntree = 2000, mtry = 6) to obtain the importance of input variables and screen out the top five genes (Fig. 2C–D). The SVM-RFE algorithm was used to remove the last few feature genes in the weight ranking of the training set in one round, and 22 genes were screened (Fig. 3A). According to the XGBoost model (Fig. 3B), 19 genes were screened and showed good discrimination, with an AUC of 0.964 (Fig. 3C). Finally, the recursive feature elimination in the ‘caret’ package was used to construct different models. The results showed that lrFuncs screened 15 genes (Fig. 4A), IdaProfile screened 26 genes (Fig. 4B), caretFuncs screened 12 genes (Fig. 4C), and nbFuncs screened 24 genes (Fig. 4D).

**Fig. 5:**
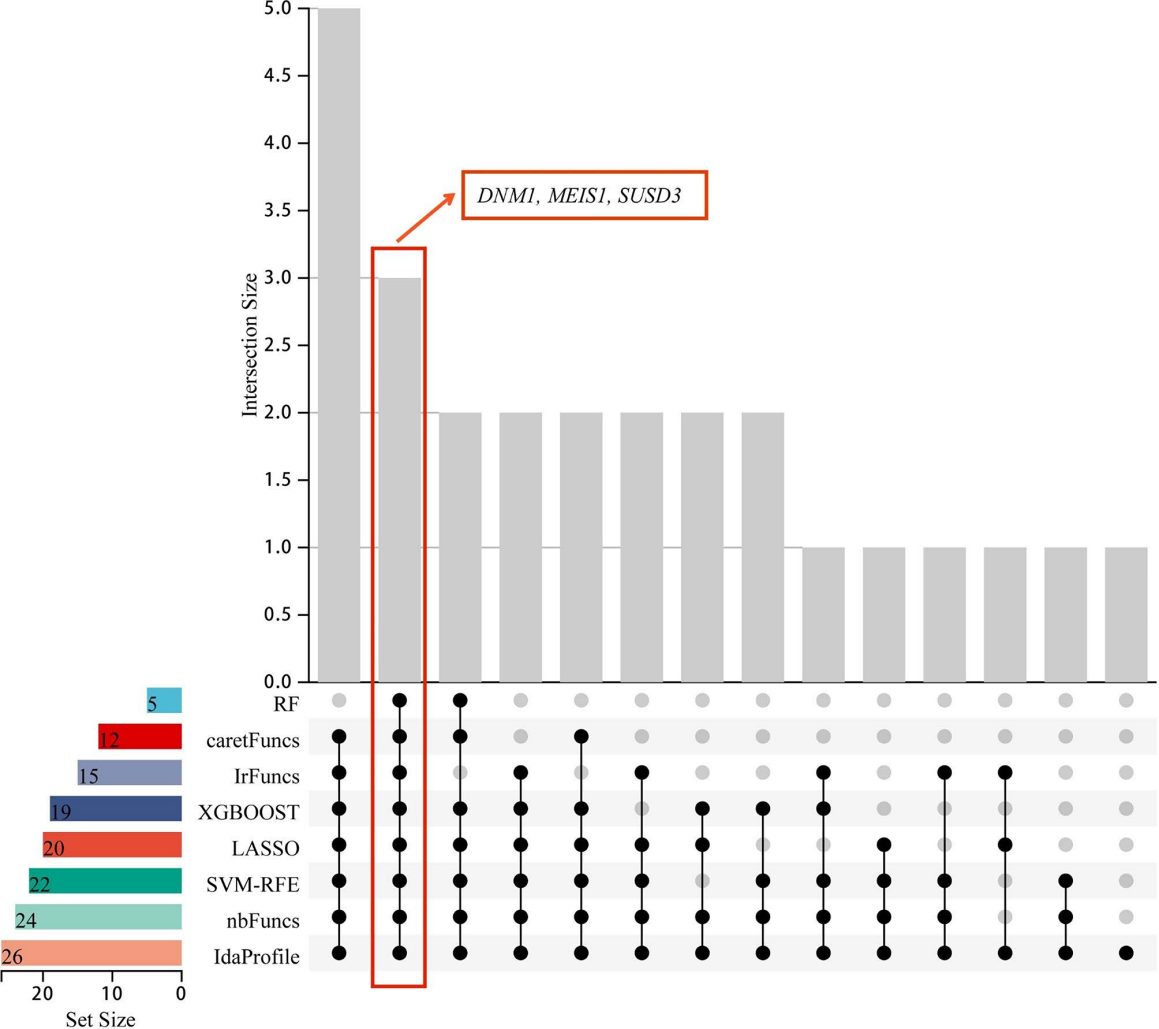
The distribution of important genes screened by each machine learning. The lower left band shows the number of important genes contained in each machine learning type. The points and lines in the lower right corner represent a subset of machine learning events. The eight dots and lines connected simultaneously indicate the common intersection of these eight machine learning events. The number of related genes in each subset is represented in the histogram

The important genes screened by each machine learning algorithm have been obtained (Table 2). And the intersection genes of important genes screened by these machine-learning algorithms are also summarized in Table 2. Figure 5 is the Upset diagram, which illustrating shared genes in important genes screened by different machine learning algorithm. The results showed that DNM1, MEIS1, and SUSD3 can be regarded as key genes for subsequent studies.

**Table 2:** The results of all machine learning algorithms used for screening important genes related to AML prognosis

Items	Machine learning algorithms								Intersection genes
	LASSO	RF	SVM	XGBOOST	IdaProfile	nbFuncs	caretFuncs	lrFuncs	
Important genes	TFF3	TFF3	TFF3	TFF3	TFF3	TFF3	TFF3	HK3	DNM1
	CTSE	HOXA7	SUSD3	HOXA7	SUSD3	SUSD3	HOXA7	CTSE	SUSD3
	SLC25A21	MEIS1	S100P	MEIS1	S100P	S100P	DNM1	PF4	MEIS1
	BMX	SUSD3	CTSE	CTSE	CYP4F2	CYP4F2	MEIS1	SPINK2	-
	SUSD3	DNM1	CYP4F2	PF4	CTSE	CTSE	SPINK2	DNM1	-
	CYP4F2	-	BMX	DNM1	BMX	BMX	CTSE	NMU	-
	HOXA7	-	SLC25A21	VNN2	SLC25A21	SLC25A21	SLC25A21	S100P	-
	DNM1	-	DNM1	SUSD3	DNM1	DNM1	BMX	FGF13	-
	FGF13	-	HOXA7	RFESD	C17orf99	C17orf99	CYP4F2	EPB42	-
	C17orf99	-	C17orf99	HOXA5	HOXA7	HOXA7	HOXA5	CYP4F2	-
	CA3	-	RFESD	SLC25A21	LIN7A	LIN7A	SUSD3	CLEC5A	-
	SPINK2	-	SPINK2	CYP4F2	RFESD	FGF13	S100P	SUSD3	-
	S100P	-	LIN7A	RTN1	FGF13	RFESD	-	MEIS1	-
	MEIS1	-	FGF13	SPINK2	HK3	HK3	-	CA3	-
	NKX2.3	-	HK3	BMX	SPINK2	SPINK2	-	BMX	-
	RTN1	-	CA3	HK3	CA3	CA3	-	-	-
	CLEC5A	-	MEIS1	NKX2.3	CLEC5A	CLEC5A	-	-	-
	PF4	-	HOXA5	CA3	MEIS1	MEIS1	-	-	-
	HOXA5	-	CLEC5A	S100P	NMU	NMU	-	-	-
	EPB42	-	NMU	-	HOXA5	HOXA5	-	-	-
	-	-	VNN2	-	VNN2	VNN2	-	-	-
	-	-	PF4	-	PF4	PF4	-	-	-
	-	-	-	-	RTN1	RTN1	-	-	-
	-	-	-	-	NKX2.3	NKX2.3	-	-	-
	-	-	-	-	EPB42	-	-	-	-
	-	-	-	-	IL1R2	-	-	-	-

AML: acute myeloid leukemia

### Pan-cancer analysis for key genes associated with AML prognosis

**Fig. 6:**
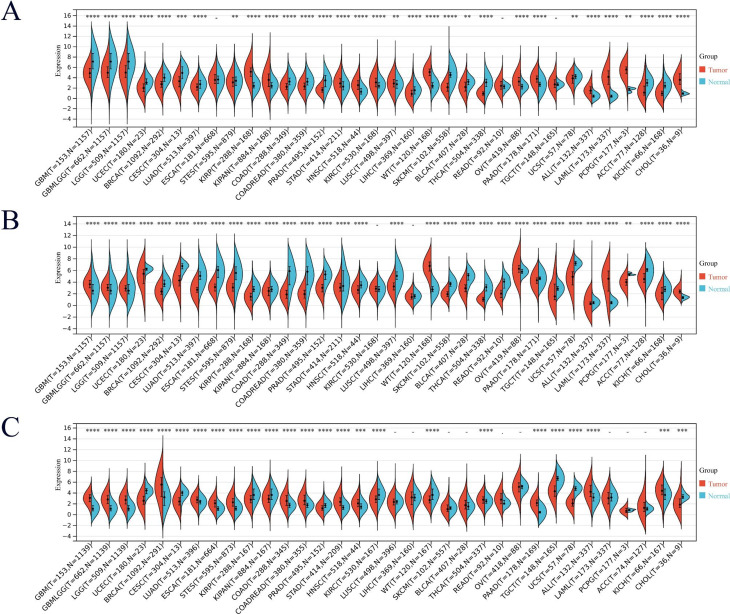
MEIS1, DNM1, and SUSD3 gene expression levels in cancers based on TCGA and GTEx. (**A**) MEIS1 gene expression levels. (**B**) DNM1 gene expression levels. (**C**) SUSD3 gene expression levels. N: Normal tissues; T: Tumor tissues; Numerical values pertaining to N and T indicate the sample size of normal or tumor tissue in different cancer types. *p < 0.05; **p < 0.01; ***p < 0.001

For the three key genes screened by machine learning tools, single gene pan-cancer analysis was performed respectively. The results showed that DNM1, MEIS1, and SUSD3 were differentially expressed between various cancers and normal tissues. MEIS1 and DNM1 are highly expressed in AML (Fig. 6A and B), while SUSD3 is not significantly different in AML (Fig. 6C).

**Fig. 7:**
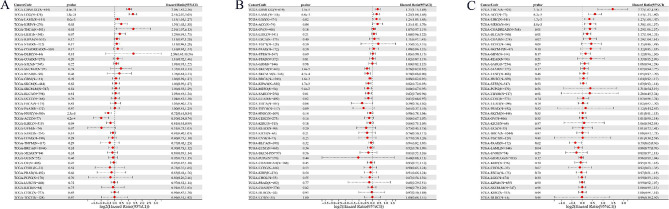
Forest plot for the overall survival prognostic analysis of MEIS1, DNM1, and SUSD3 gene expression in human cancers based on TCGA and GTEx. (**A**) The correlation between the expression levels of MEIS1 genes and the prognosis of various cancers. (**B**) The correlation between the expression levels of SUSD3 genes and the prognosis of various cancers. (**C**) The correlation between the expression levels of DNM1 genes and the prognosis of various cancers

To study the relationship between the expression levels of DNM1, MEIS1, and SUSD3 and the prognosis of 33 kinds of cancer, we carried out a survival correlation analysis. Cox proportional hazard model analysis showed that MEIS1 was not only associated with poor prognosis of AML (*p* = 8.0e-3, HR = 1.15), but also associated with poor prognosis of GBML, LGG, KIRP, and THCA (Fig. 7A). At the same time, MEIS1 was significantly correlated with the prognosis of HNSC, ACC, and KIRC. In addition to the poor prognosis of AML (*p* = 6.6e-3, HR = 1.25), SUSD3 was also significantly associated with the poor prognosis of GBML, LGG, and ACC (Fig. 7B). At the same time, SUSD3 was significantly associated with a better prognosis of various types of cancer (SKCM, SKCM-M, BRCA, KIPAN, MESO, SARC, LUAD). DNM1 was significantly associated with a better prognosis of AML (*p* = 0.04, HR = 0.88) and PAAD (*p* = 0.02, HR = 0.79) (Fig. 7C). In addition, DNM1 was also significantly associated with poor prognosis in a variety of types (THCA、ACC、LIHC、MESO、COADREAD、BLCA、COAD).

**Fig. 8:**
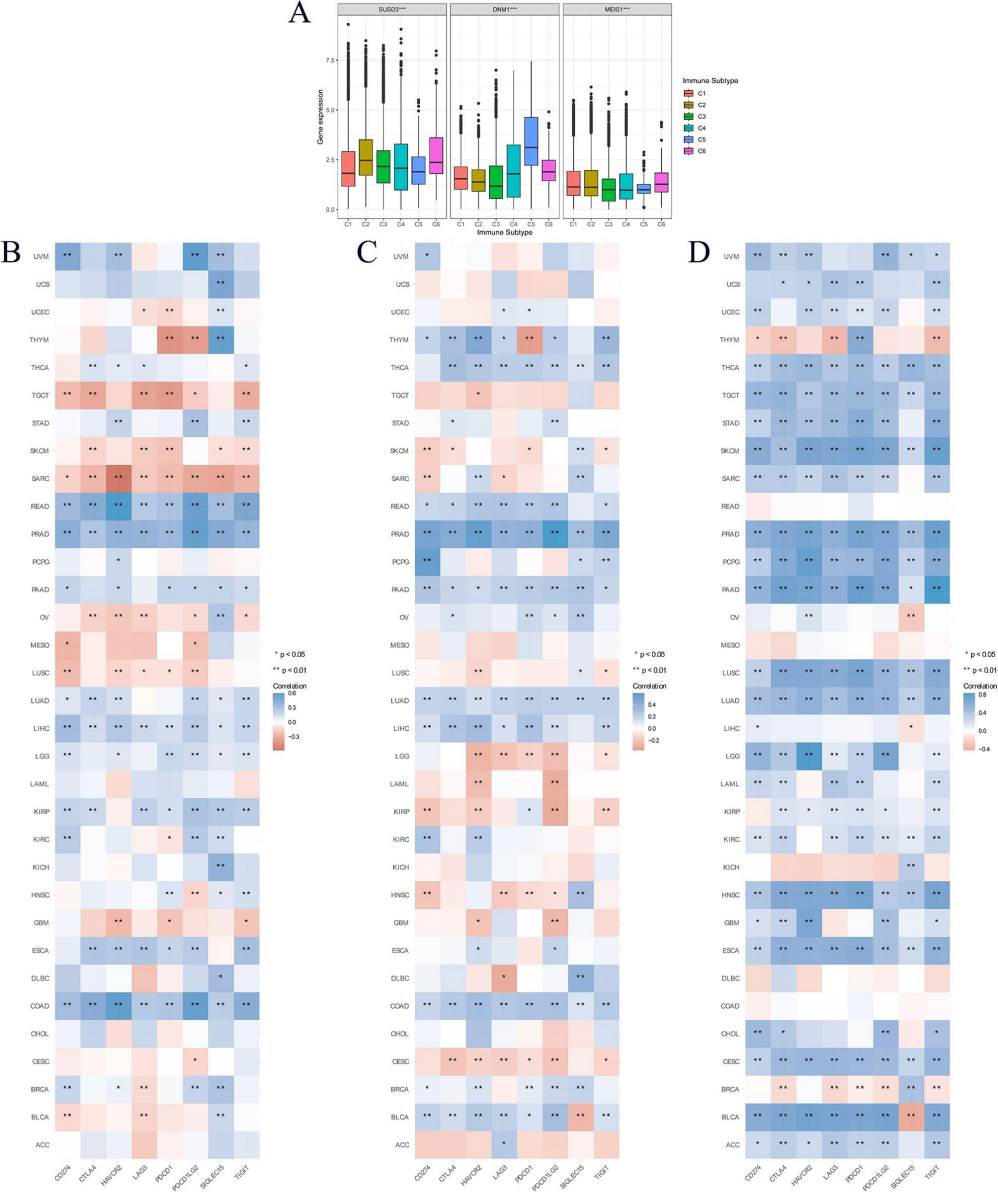
Correlation analysis between gene expression level and immune subtypes or immune checkpoint genes in pan-cancer (**A**) The relationship between MEIS1, DNM1, and SUSD3 expression and pan-cancer immune subtypes. (C1, wound healing; C2, IFN-gamma dominant; C3, inflammatory; C4, lymphocyte depleted; C5, immunologically quiet; C6, TGF-b dominant) (**B**) The relationship between MEIS1 expression and pan-cancer immune checkpoint genes. (**C**) The relationship between DNM1 expression and pan-cancer immune checkpoint genes; (**D**) The relationship between SUSD3 expression and pan-cancer immune checkpoint genes. *P < 0.05; **P < 0.01; ***P < 0.001

To explore the relationship between the expression levels of key genes and AML immune subtypes, we analyzed the correlation between key genes and AML immune subtypes based on the TCGA data set. The results showed that DNM1 (*p* < 0.001), MEIS1 (*p* < 0.001), and SUSD3 (*p* < 0.001) were significantly associated with AML C1-C6 immune subtypes (Fig. 8A). Based on the TCGA database, the correlation between key gene expression levels and immune checkpoints was explored. The results showed that the expression levels of DNM1, MEIS1, and SUSD3 were associated with many cancer immune checkpoints (Fig. 8B–D). Specifically, the expression level of MEIS1 was not correlated with the AML immune checkpoint (Fig. 8B). Figure 8C shows that the expression level of DNM1 is significantly correlated with the two immune checkpoints of AML (HAVCR2 and PDCD1LG2). Figure 8D showed that the SUSD3 expression level was significantly correlated with the five immune checkpoints of AML (CD274, CTLA4, LAG3, PDCD1, and TIGIT).

### Gene set enrichment analysis (GSEA) of key genes

**Fig. 9:**
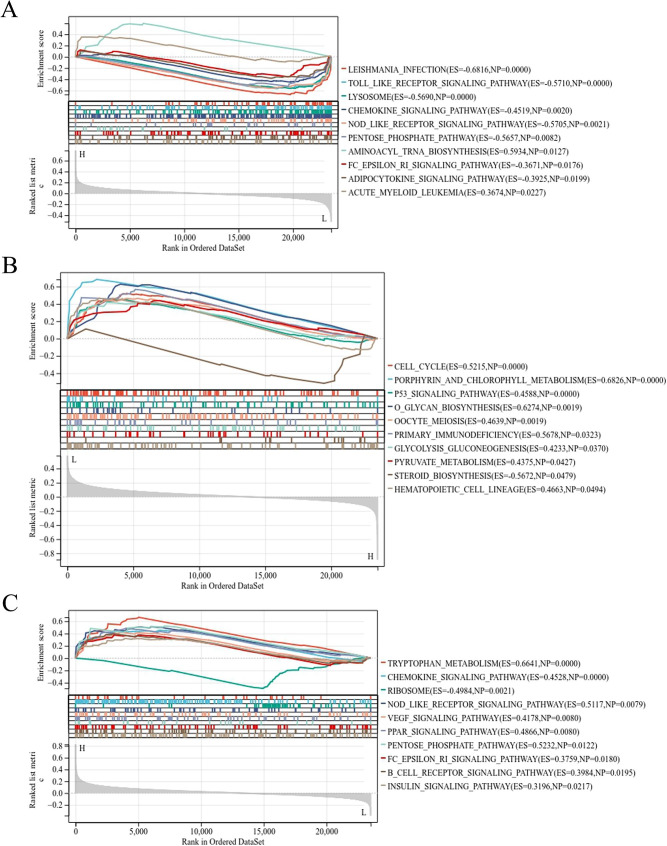
Gene Set Enrichment Analysis (GSEA) for key genes. (**A**) the potentially enriched pathways related to genes, which are closely associated with DNM1 in the training set (GSE15061); (**B**) the potentially enriched pathways related to genes, which are closely associated with MEIS1 in the training set; (**C**) the potentially enriched pathways related to genes, which are closely associated with SUSD3 in the training set

GSEA analysis showed that genes related to DNM1 in the training set were mainly enriched in aminoacyl tRNA biosynthesis, acute myeloid leukemia and other pathways (Fig. 9A). Genes related to MEIS1 were mainly enriched in pathways such as cell cycle, P53 signaling pathway, o glycan biosynthesis, etc. (Fig. 9B). Genes associated with SUND3 are mainly enriched in tryptophan metabolism, NOD-like receptor signaling pathway, chemokine signaling pathway etc. (Fig. 9C).

### Validation of diagnostic value of key genes

**Fig. 10:**
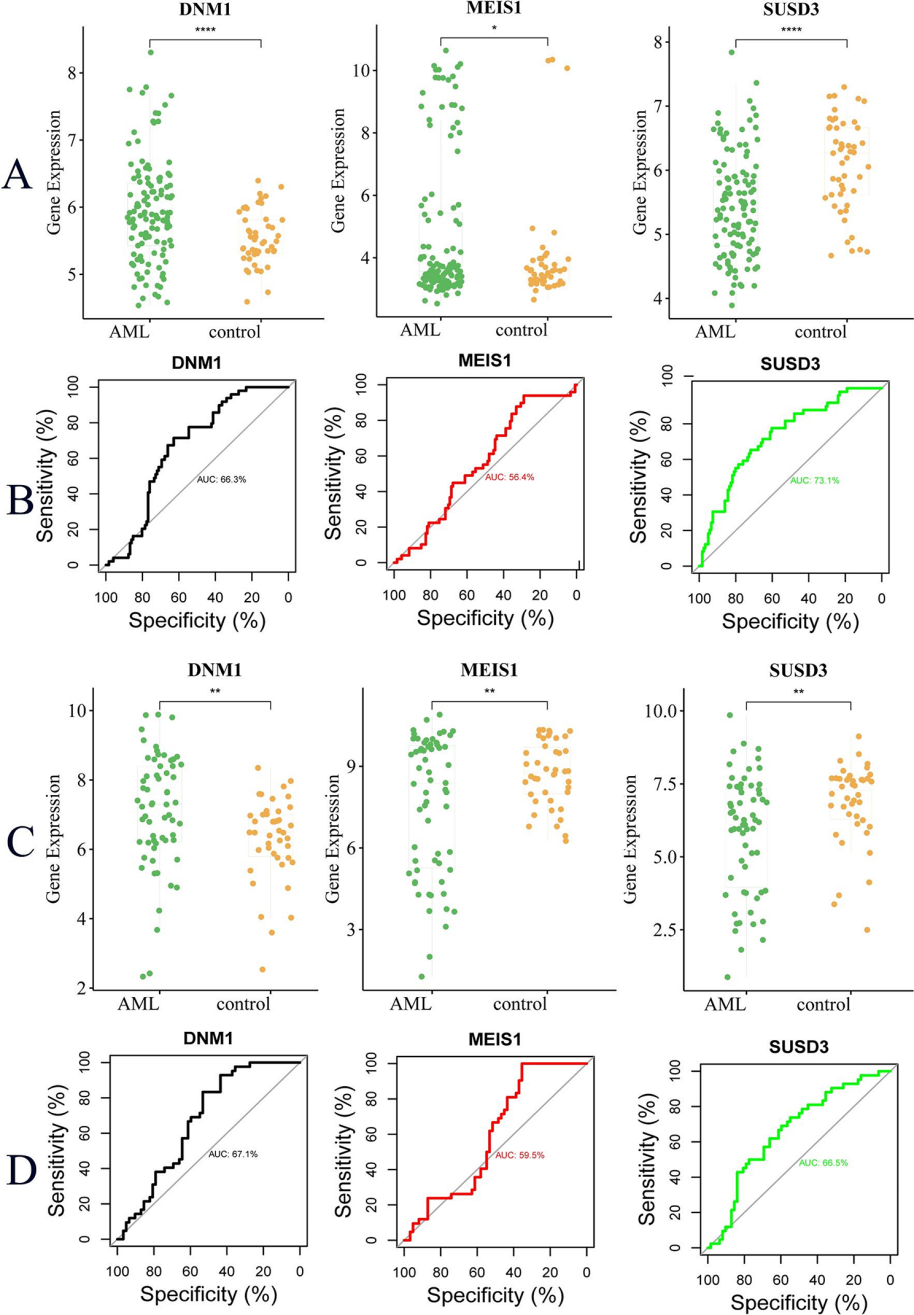
Expression difference analysis and diagnostic value assessment of MEIS1, DNM1, and SUSD3 in the validation set. (**A**) Analysis of differences in the expression of three genes in dataset GSE48588; (**B**) Assessment of the diagnostic value of three genes in dataset GSE48588; (**C**) Analysis of differences in the expression of three genes in dataset GSE63270; (**D**) Assessment of the diagnostic value of three genes in dataset GSE63270. *P < 0.05; **P < 0.01; ***P < 0.001

To further explore the role of key genes as AML biomarkers, we selected two data sets to verify their diagnostic value. The results showed that in the GSE48588 (Fig. 10A–B) and GSE63270 (Fig. 10C–D) datasets, the expression levels of DNM1 and MEIS1 in AML were significantly higher than those in the control group, while the expression levels of SUSD3 in AML were significantly lower than those in the control group. ROC results suggested that the expression differences of DNM1, MEIS1, and SUSD3 have potential diagnostic value for AML.

## Discussion

The prognosis of AML is poor. Young and elderly patients have a high risk of recurrence of chemotherapy resistance, and alternative and targeted drugs are needed to improve their survival rate [22]. At present, the treatment of AML mainly includes chemotherapy and molecular targeted therapy, such as FMS-like tyrosine kinase 3 (FLT3) inhibitors, IDH [isocitrate dehydrogenase (NADP+)] inhibitors, and monoclonal antibodies [23]. Despite many treatments, the prognosis of AML is still poor. High-throughput genomic screening methods and computer-aided techniques can be used to predict biomarkers related to disease occurrence and assist in the design of new targeted drugs [24]. Therefore, screening biomarkers related to the prognosis of AML patients through machine learning methods will provide a valuable reference for individualized targeted therapy and prognosis prediction of AML in clinical practice.

In this study, 26 genes with survival differences were screened from the differentially expressed genes in the training set. To further screen out the key genes closely related to the prognosis of AML, this study screened key genes based on a variety of machine learning. The results showed that the machine learning method used in this study identified DNM1, MEIS1, and SUSD3 as key genes significantly associated with AML prognosis based on different screening criteria. To further clarify the correlation between key genes and the occurrence and development of AML and various cancers, this study also performed a single-gene pan-cancer analysis of three key genes based on the TCGA database. The expression of MEIS1 and DNM1 in AML and normal controls was significantly different. MEIS1, SUSD3, and DNM1 were significantly associated with the prognosis of AML patients. Three key genes were significantly associated with AML immune subtypes, and DNM1 and SUSD3 were significantly associated with multiple immune checkpoints of AML. In addition to the strong association between the three key genes and AML, this study also found evidence that they are closely related to the prognosis and immunity of various cancers. More importantly, we also selected two validation datasets to verify the diagnostic value of key genes for AML, and the results showed that DNM1, MEIS1, and SUSD3 had good diagnostic values for AML.

The myeloid tropism leukemia virus integration site 1 (MEIS1) gene is located on 1p13-14 of human chromosome 2 and is widely expressed in various tissues including blood, liver, and brain [25]. MEIS1 is related to the differentiation of leukemia stem cells and the proliferation of leukemia cells [26]. Studies have shown that MEIS1 is often up-regulated in AML patients and can participate in disease progression through a variety of mechanisms [27, 28]. Thorsteinsdottir, U. et al. highlighted the role of Meis1 in regulating human AML cell maintenance and survival in vitro knockdown experiments [29]. Similar to the above study is that our study has also found evidence that MEIS1 expression is associated with AML prognosis, immunity, etc., which further proves that MEIS1 may be a biomarker for predicting AML prognosis.

Dynamins 1 (DNM1) is a member of the GTP-binding protein family. DNM1 is highly expressed in the nervous system of the human body and can regulate nerve activity [30, 31]. Therefore, DNM1 is often reported to play a role in nervous system diseases [32, 33]. However, in addition to neurological diseases, more and more studies have shown that DNM1 plays a role in the development of many cancers [34–36]. Previous studies have found that high expression of DNM1 is an independent prognostic biomarker for poor OS in patients with hepatocellular carcinoma [36]. DNM1 is overexpressed in many lung cancers, enhances the growth, migration, and invasion of cancer cells, and reduces the survival rate of lung cancer patients. Activated DNM1 selectively regulates tumor necrosis factor-related apoptosis-inducing ligand (TRAIL-R2) -mediated endocytosis, allowing cancer cells to escape death [37]. Based on the above, DNM1 may be used as a biomarker to predict the prognosis of patients with multiple cancers. In this study, we found for the first time evidence that DNM1 is potentially related to the prognosis of AML patients, further indicating that DNM1 plays a potential role in the occurrence and development of AML, and the specific mechanism of action is worthy of further discussion.

At present, there are few reports on Sushi domain-containing protein 3 (SUSD3), mainly focusing on the mechanism of SUSD3 in the occurrence and development of breast cancer. SUSD3 has extracellular, transmembrane, and cytoplasmic domains. It is highly expressed in breast cancer and estrogen-sensitive tissues such as the liver, breast, myometrium, endometrium, and ovary. Experiments have shown that SUSD3 has a higher level of expression in estrogen receptor (ER) -positive breast cancer cells, and estrogen treatment can further increase its expression [38]. SUSD3 has been reported as one of the potential biomarkers for the prognosis of breast cancer [39, 40]. This study found for the first time that SUSD3 is potentially related to the prognosis of AML and is expected to be a prognostic marker for AML patients.

In addition, we explored potential pathways associated with genes closely related to key genes in the training set through GSEA. Many pathways were found to be potentially related to the development of AML. Previous study has reported that the dysfunction of the typical metabolomics pathway Aminoacyl − tRNA biosynthesis indicates that mitochondrial dysfunction, which leads to a decrease in the detoxification ability of reactive oxygen species produced by AML chemotherapy and radiotherapy [41]. P53 plays a key role in normal and leukemia hematopoiesis and is the core of the complex network of AML-related signaling pathways [42]. NLRP3 inflammasome, a major factor in NOD-like receptor signaling pathway, promotes the progression of AML in an IL-1β-dependent manner. Targeting NLRP3 inflammasome may provide a new therapeutic option for AML [43]. Based on the above, it can be seen that the potential pathways related to DNM1, MEIS1 and SUSD3 participate in the occurrence and development of AML. We hypothesized that DNM1, MEIS1, and SUSD3 are closely related to the prognosis of AML, which may be mediated by the above pathways. However, the above is only speculation, and further functional verification experiments are needed to explore the mechanism of these three key genes in the occurrence and development of AML.

Based on a variety of machine learning, this study has explored three new biomarkers for AML prognosis, which provides a new idea for the clinical development of individualized targeted therapy and prognosis prediction of AML. However, this study still has some shortcomings. First, all the analyses in this study are based on retrospective data in public databases, and large-scale prospective studies and additional functional verification experiments are needed to confirm our findings. Secondly, it is necessary to further explore the specific mechanism of the three key genes in AML and their influence on the prognosis of AML in future research, so as to better explore the molecular mechanism involved in tumorigenesis and AML development.

## Conclusion

This study found that DNM1, MEIS1, and SUSD3 were abnormally expressed in AML and were potentially related to its prognosis. They are expected to become new biomarkers and potential therapeutic targets for predicting the prognosis of AML patients. This study will provide a new theoretical basis for the basic research of AML.

### Electronic supplementary material

Below is the link to the electronic supplementary material.

Supplementary Material 1

## Data Availability

The datasets generated and/or analyzed during the current study are available in the [GEO] repository, [GSE63270, GSE15061, and GSE48558].
