# Supplementary material for: Multiple machine-learning tools identifying prognostic biomarkers for acute Myeloid Leukemia
Source: BMC Med Inform Decis Mak. 2024 Jan 2;24:2. doi: 10.1186/s12911-023-02408-9 (PMC10759623; doi:10.1186/s12911-023-02408-9)
Supplement: Supplementary file 1 — Supplementary Material 1 [file 12911_2023_2408_MOESM1_ESM.docx]

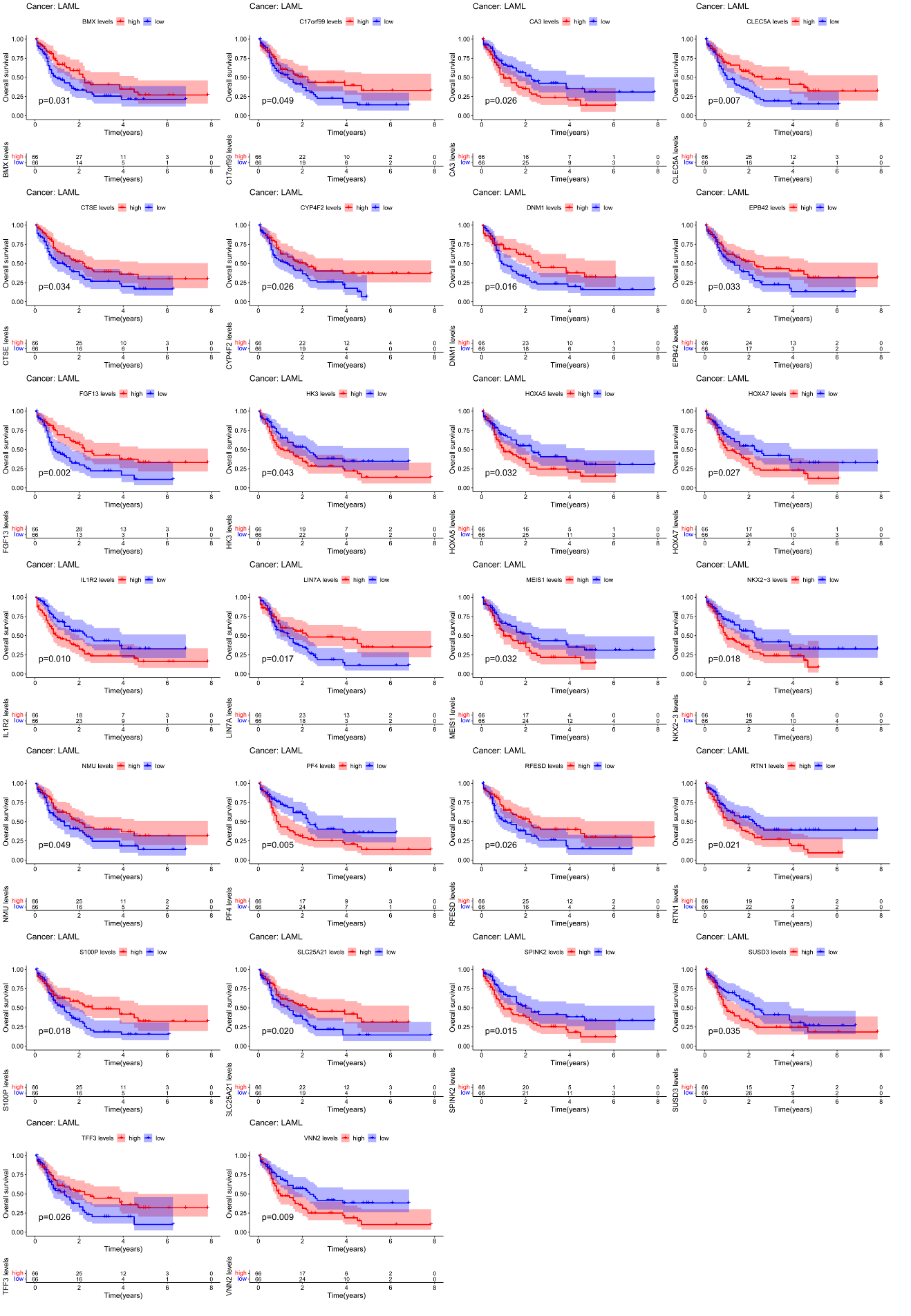


**Supplemental Figure 1** KM survival analysis of OS and DFS between high- and low-expression groups of genes associated with AML prognosis in training sets GSE15061.
